# Day‐to‐Day Reproducibility of Arterial Stiffness Following a Bout of Resistance Exercise in Healthy Young Females: A Reliability Study

**DOI:** 10.1002/hsr2.71688

**Published:** 2025-12-29

**Authors:** Wesley Tyler Blumenburg, Kayla M. Soave, Katharine Dianne Currie

**Affiliations:** ^1^ Department of Kinesiology Michigan State University East Lansing Michigan USA

**Keywords:** acute exercise, blood pressure, pulse wave analysis, reproducibility of results

## Abstract

**Background and Aims:**

Aerobic and resistance exercise (RE) are both promoted as positive lifestyle modifications to improve cardiovascular health. While research has shown that regular aerobic exercise can improve arterial stiffness, paucity remains surrounding the vascular effects of RE. Acute RE is shown to increase and decrease arterial stiffness following exercise bouts. Additionally, the day‐to‐day reliability of post‐RE arterial stiffness responses are currently unknown.

**Purpose:**

To understand the day‐to‐day reliability of arterial stiffness following two identical bouts of acute RE in healthy young females.

**Methods:**

Fifteen non‐resistance trained females (21 ± 3 years) completed two 60‐min full body RE sessions with a 7‐min aerobic warm‐up and cooldown on separate days. For each session, arterial stiffness (i.e., carotid‐femoral pulse wave velocity [cfPWV]) was measured before and for an hour post‐RE in 15‐min intervals. Test‐retest reliability of pre‐ and post‐RE cfPWV from the two visits was assessed using intraclass correlation coefficients (ICC) and a linear mixed model (LMM).

**Results:**

cfPWV revealed good reliability at rest (ICC: 0.87, 95% confidence intervals [CI]: 0.66–0.96) and post‐RE at 15 (ICC: 0.76, 95% CI: 0.43–0.91), 30 (ICC: 0.72, 95% CI: 0.35–0.90), and moderate reliability at 45 (ICC: 0.69, 95% CI: 0.29–0.88), and 60 min post‐RE (ICC: 0.71, 95% CI: 0.33–0.90). LMM analysis demonstrated good reliability for cfPWV across visits (ICC: 0.81), with no significant main effect for visit (F (1,126) = 0.308, *p* = 0.580) or visit × time interaction (F (4,126) = 0.312, *p* = 0.869), indicating consistent temporal patterns between RE bouts. However, a significant main effect for time was observed (F (4,126) = 4.10, *p* = 0.004).

**Conclusion:**

These findings suggest that cfPWV measurements at rest and following an acute bout of RE demonstrate moderate to good day‐to‐day reliability in healthy young females for up to an hour, supporting the use of cfPWV in research investigating vascular responses to RE.

## 1 Introduction

Increased arterial stiffness, either with aging or poor lifestyle habits such as physical inactivity, is associated with an increased risk of cardiovascular disease (CVD) and cardiovascular mortality [1]. In order to improve health and reduce the risk for CVD and all‐cause mortality, public health guidelines advocate for regular aerobic and resistance exercise (RE) [2, 3, 4]. As evidenced by meta‐analyses, aerobic exercise is well established to decrease arterial stiffness both acutely (i.e., following a single bout of aerobic exercise) [5] and chronically (i.e., following exercise training) [6]. Conversely, the effects of both acute and chronic RE on the vascular system appear unclear [7, 8]. Given that acute physiological responses to a single bout of aerobic exercise have been shown to predict the magnitude of change in the physiological outcome following chronic training [9, 10, 11], the equivocal responses to RE warrant additional investigation.

In 2007, Heffernan and colleagues found that a single bout of RE consisting of eight exercises for three sets of ten repetitions significantly increased arterial stiffness, measured via carotid‐femoral pulse wave velocity (cfPWV), in healthy adult males [12]. More recently, using a similar RE protocol and population, Pierce et al. found cfPWV did not change for up to an hour following an acute RE bout [13]. Despite having similar exercise prescriptions, these inconsistent findings may be attributed to differences in measurement techniques/systems [14] or day‐to‐day variations in cfPWV in response to exercise. While resting cfPWV has been shown to have good day‐to‐day reliability [15], it is presently unknown if acute post‐RE measurements show the same consistency. Establishing this is paramount to study designs investigating the effect of RE and arterial stiffness. Furthermore, research examining acute cfPWV responses to RE have predominantly focused on males [12, 16, 17, 18, 19, 20] and mixed samples [21, 22, 23, 24, 25], with limited studies analyzing females individually [26, 27, 28]. A paucity of findings among females highlights a need for investigations focusing on vascular responses to RE in female populations.

The primary aim of this study was to determine if arterial stiffness responses following a single bout of RE demonstrate day‐to‐day reliability in healthy young females. We hypothesized that cfPWV, a gold‐standard measure of arterial stiffness, would exhibit moderate test‐retest reliability following two acute bouts of RE. Establishing the consistency of these acute vascular responses is essential for improving research on RE and its effects on cardiovascular health in females.

## 2 Methods

All procedures involving human participants were approved by the Michigan State University Biomedical and Health Institutional Review Board (Project reference number: 00005466). The study was performed in accordance with the ethical standards of the declaration of Helsinki. All participants provided written consent prior to participation.

Twenty females between the age of 18–35 years. were enrolled in the study between 2021 and 2023. Due to loss to follow‐up, 15 participants were included in the analysis. All participants did not participate in regular resistance training, defined as 2 days of RE a week, within 6 months of participating in the study. Participants were also free of any physical limitations that may impact their ability to exercise, prior diagnosis of hypertension or cardiovascular disease, diabetes, and pregnancy.

### 2.1 Study Design

This study consisted of three visits. The initial visit included written informed consent followed by the completion of two questionnaires to determine eligibility. The Physical Activity Readiness Questionnaire for Everyone (PAR‐Q+) was administered to assess the participants readiness and eligibility to exercise [29]. If eligible, a heath history and demographics questionnaire was given. This questionnaire contains questions about general demographics, health history, medications, and physical activity habits. Once eligibility was confirmed, anthropometric measurements were then obtained. Maximal upper and lower body muscular strength was determined by performing a 1‐repetition max (1‐RM) test via bench press and leg press, respectively. 1‐RM testing has demonstrated good day‐to‐day reliability for both upper and lower body assessments regardless of training status and sex [30]. Prior to 1‐RM testing, investigators provided orientation on exercise equipment and proper exercise technique for each movement. Participants started by performing a single repetition around 50% of their perceived maximal weight based on self‐reported training history. The 1‐RM was achieved within four trials. Weight was increased by 2–20 kg each repetition until the participant was unable to complete the selected repetition. The final weight lifted successfully was recorded as their absolute 1‐RM [31].

Visits two and three consisted of identical procedures. All testing was performed in a temperature controlled and dimly lit room. Study visits were scheduled at the same time of day for each participant with at least 24 h in between visits. RE visits were scheduled, an average of 8 ± 4 days apart; there was no control for phase of menstrual cycle or hormonal contraceptive use between visits. Participants were instructed to abstain from vigorous exercise for 24 h, caffeine and alcohol consumption for 12 h, and food, drink, and nicotine (i.e., smoking vaping or chewing) products for 3 h prior to each visit.

Following 10 min of quiet rest in the supine position, baseline cfPWV and blood pressure (BP) measurements were taken. BP measurements were taken from the right arm positioned at heart level at 1‐min intervals, utilizing an automated device (Carescape V100; GE Healthcare, Milwaukee, WI, USA). Resting (i.e., pre‐ex) BP and heart rate values were calculated as the average of three measurements with a systolic BP within 5 mmHg of each other [32, 33].

Following resting measurements, participants were fitted with a Polar H10 heart rate strap (Polar H10; Polar Electro Oy, Kempele, Finland). Participants then performed a full‐body RE workout based on the recommendations of the American College of Sports Medicine (ACSM) which included seven exercises targeting all major muscle groups (bench press, shoulder press, leg press, leg curl, row, bicep curl, and triceps extension), and a brief warm‐up and cooldown [31]. The warm‐up involved 7 min of treadmill walking at 50% of heart rate reserve; maximal heart rate was estimated using 220‐age and resting HR was taken from the BP measurements. In line with ACSM guidelines for RE, each exercise was prescribed at 60%–80% 1‐RM for three sets of 8–12 repetitions [34]. For each exercise, a weight was selected so that the last few repetitions of each set were difficult to complete while maintaining proper technique. The same load and repetitions for all exercises were matched on both RE visits. Rest of 2 min was given between each exercising set. Directly after completion of the RE bout, participants completed a brief 7 min cooldown consisting of walking at the participant's selected speed.

Immediately following the cooldown, participants returned to the supine position where they rested quietly for 60 min; post‐exercise (post‐RE) assessments of cfPWV and BP (duplicate measures) were captured at 15, 30, 45 and 60 min post‐ex.

### 2.2 cfPWV Assessment

cfPWV measurements were collected in accordance with the most recent guidelines [33]. All cfPWV measures were obtained by the same trained investigator (KDC) who has demonstrated day‐to‐day reliability in other populations [35, 36]. All cfPWV measurements were taken from sequential recordings of carotid and femoral arterial pressure waveforms obtained from the right side of the body using a handheld tonometer (Model SPT‐301; Millar Instruments Inc., Houston, TX). Participants were also instrumented with a single‐lead electrocardiogram (ECG) to collect heart rate during the measurements (Model ML‐132; ADInstruments Inc.). A minimum of 30 s of arterial pressure waveforms were collected at each site using propriety data collection equipment and software (Labchart 8; ADIntstruments Inc., Colorado Springs, CO, USA). The arterial pressure waveforms were subjected to band‐pass filtering (2–30 Hz), and the arrival of the waveform at each site was determined as the minimum value of the filtered signal. Subsequently, a 10 s sample of waveforms was analyzed, and the time from the start of cardiac cycle to the arrival of the waveform at each site was calculated; the R‐spike of the ECG signal was used to indicate the start of the cardiac cycle. Pulse transit time (Δt) was then calculated as the difference between the values calculated at the femoral and carotid sites. The direct distance (D) between the carotid and femoral sites was measured along the body's surface using anthropometric tape. cfPWV was then calculated as (0.8 × D)/Δt. cfPWV measurements were analyzed in duplicate and averaged for analysis. In instances where the difference between cfPWV values was > 0.5 m·s^−1^, a third 10 s section was analyzed, and the median cfPWV value was reported [37].

### 2.3 Statistical Analyses

A sample size calculation was conducted a priori, with predetermined type 1 error and power set at 0.05 and 0.80, respectively. To achieve an intraclass correlation coefficient (ICC) of 0.70, a sample size of 15 was deemed sufficient [38]. Test‐retest reliability of pre‐ and post‐RE cfPWV and systolic BP from the two sessions was assessed using ICC (ICC, model 1, 1) and a linear mixed effect model (LMM). ICC reliability was defined and presented as ICC with 95% confidence intervals (CI). Reliability was categorized as poor for an ICC of ≤ 0.51, moderate between 0.51 and 0.75, good between 0.76 and 0.90, and excellent ≥ 0.91 [39]. Systolic BP was included in the analysis due to its close association with cfPWV.

The ICC was used to evaluate the relative reliability of these measures by quantifying the degree of agreement between sessions, providing an estimate of the agreement in individual rankings across visits. A LMM was employed to examine whether systematic differences existed between visits and to model the course of cfPWV over time post‐RE, as LMMs are suited for analyzing repeated measured data for test‐retest reliability [40]. The LMM included fixed effects for time (baseline, 15, 30, 45, and 60 min) and visit (second vs. third visit) to assess whether responses differed across sessions. A random intercept for subjects was incorporated to account for individual baseline differences and within‐subject correlations. Model diagnostics included visual inspection of residual normality using Q–Q plots and histograms. Statistical analyses for ICC's were calculated using SPSS software (version 26; IBM Corporation, Armonk, NY, USA); LMMs were performed using jamovi (version 2.0.0.0).

## 3 Results

Fifteen females (21 ± 3 years.) completed the study with a mean body mass index of 19.2 ± 4.5 kg/m^2^. The mean time between RE bout 1 and 2 was 8 ± 5 days of each other. Of the sample, 100% identified as non‐Hispanic with 7% (1/15) identifying as Asian and 93% (14/15) as White. Sixty percent (9/15) of the sample was taking oral contraceptives, 33% (5/15) were using some form of hormonal patch, injection, or intrauterine device, and 27% (4/15) were on a natural cycle. The mean bench press and leg press 1‐RM were 35.6 ± 8.0 and 125.6 ± 45.9 kg, respectively.

**Figure 1:**
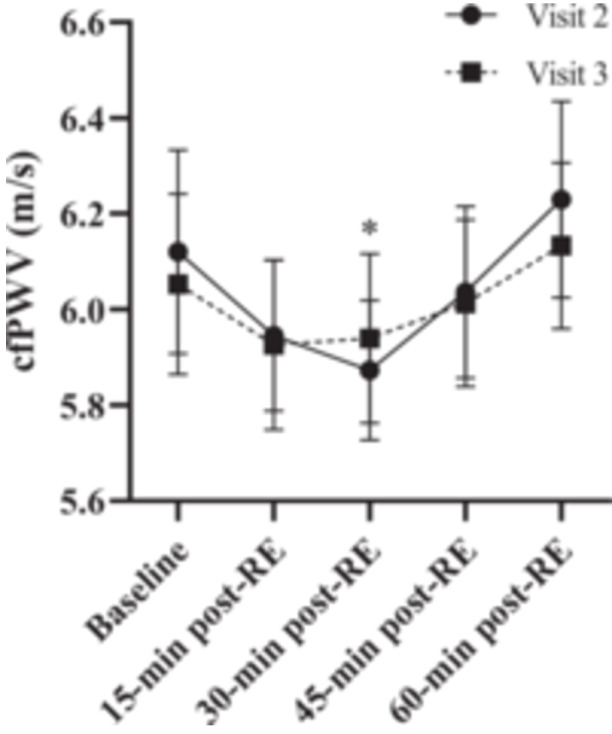
Changes in carotid‐femoral pulse wave velocity (cfPWV) following two bouts of resistance exercise (RE). Visit 2 is represented by solid lines and circles with visit 3 represented as dashed lines and squares. * Indicates a significant difference (*p* = 0.023) from baseline for both visits. Values are presented as mean ± standard error of the mean.

For cfPWV, descriptive and ICC data are presented in Table 1. All cfPWV data demonstrated a minimum of moderate test‐retest reliability (ICC between 0.51 and 0.75). LMM results demonstrated good reliability, with an ICC of 0.812, indicating cfPWV measurements were consistent and reproducible across visits. Additionally, there was no significant main effect of visit (F (1, 126) = 0.308, *p* = 0.580) indicating no systematic differences in cfPWV between visits, or interaction (F (4, 126) = 0.312, *p* = 0.869), suggesting similar temporal patterns across visits. Conversely, there was a significant main effect for time (F (4, 126) = 4.10, *p *= 0.004). cfPWV data are displayed in Figure 1.

**Table 1:** Mean values and reliability results for carotid‐femoral pulse wave velocity.

Time	Mean ± SD (m/s)		ICC	95% CI		ICC *p* value
	Visit 2	Visit 3		Lower	Upper	
Pre‐RE	6.1 ± 0.8	6.1 ± 0.7	0.873	0.664	0.955	< 0.001
Post‐RE 15 min	5.9 ± 0.6	5.9 ± 0.7	0.763	0.427	0.913	< 0.001
Post‐RE 30 min	5.9 ± 0.6	5.9 ± 0.7	0.720	0.347	0.896	< 0.001
Post‐RE 45 min	6.0 ± 0.7	6.0 ± 0.7	0.687	0.288	0.883	0.002
Post‐RE 60 min	6.2 ± 0.8	6.1 ± 0.7	0.708	0.325	0.891	0.001

Abbreviations: 95 CI, 95% confidence intervals; ICC, intraclass correlation coefficient; RE, resistance exercise.

Descriptive and ICC data for systolic BP are presented in Table 2, with most time points indicating good reliability with a minimum ICC value of 0.78. However, LMM results showed systolic BP had poor reliability, with an ICC of 0.382. Additionally, the random intercept variance was smaller than the residual variance (48.0 ± 6.93 vs. 77.7 ± 8.82), indicating increased within‐subject variability across repeated measurements. There were no significant main effects of time or visit, nor a significant interaction effect (*Ps* > 0.170), suggesting systolic BP did not systematically change across RE sessions.

**Table 2:** Mean values and reliability results for systolic blood pressure.

Time	Mean ± SD (mmHg)		ICC	95% CI		ICC *p* value
	Visit 2	Visit 3		Lower	Upper	
Pre‐RE	113 ± 12	112 ± 11	0.873	0.664	0.956	< 0.001
Post‐RE 15 min	112 ± 10	112 ± 11	0.892	0.710	0.962	< 0.001
Post‐RE 30 min	112 ± 11	112 ± 11	0.844	0.598	0.945	< 0.001
Post‐RE 45 min	113 ± 12	114 ± 11	0.780	0.461	0.920	< 0.001
Post‐RE 60 min	116 ± 12	117 ± 10	0.879	0.679	0.958	< 0.001

Abbreviations: 95 CI, 95% confidence intervals; ICC, intraclass correlation coefficient; RE, resistance exercise.

## 4 Discussion

This study is the first to investigate the test‐retest reliability of cfPWV measurements before and after acute RE in healthy young females. Our key findings demonstrate that cfPWV exhibit moderate to good test‐retest reliability both at rest (ICC = 0.87, 95% CI: 0.66–0.96) and for up to 60 min post‐RE (ICC: 0.69–0.76). Furthermore, cfPWV demonstrated good reliability across visits (ICC: 0.81) with the LMM analysis, with no significant main effect for visit or interaction between visit and time demonstrating consistent vascular responses between RE bouts for up to 60 min. The consistency of cfPWV responses across repeated RE sessions supports its utility as a robust vascular assessment in studies examining acute RE interventions.

Our findings align with prior studies reporting good reliability of resting cfPWV in healthy adults. For instance, Kimble et al. in 2019 reported good test‐retest reliability for cfPWV with an ICC if 0.83 in healthy young and middle‐aged males [41]. Furthermore, Russell et al. corroborated these findings in a mixed‐sex sample, demonstrating good test‐retest reliability for cfPWV (ICC = 0.77, 95% CI: 0.61–0.87) [42]. In agreement with prior research, our findings confirm the test‐retest reliability of resting cfPWV, expanding upon previous findings by providing sex‐specific reliability estimates in young health females. Furthermore, post‐RE cfPWV demonstrated reliability ranging from moderate to good for up to an hour post exercise. To date, this is the only study to investigate cfPWV test‐retest reliability following an acute bout of RE. The observed reliability of post‐RE cfPWV is comparable to that of aerobic exercise. Perissiou and colleagues in 2019 demonstrated that cfPWV at rest and 10 min post‐moderate intensity aerobic exercise exhibited excellent and good reliability in older adults with an ICC of 0.91 and 0.87, respectively [43].

The LMM revealed a significant main effect of time, indicating that cfPWV changed across time points during both visits. Importantly, there was no significant main effect of visit and no significant visit × time interaction, suggesting that both the overall magnitude and the temporal pattern of responses were consistent across visits. Out findings support the stability and reproducibility of cfPWV responses at rest and post‐RE in females, indicating acceptable test‐retest reliability. Although the current investigation was not explicitly designed to examine directional changes in cfPWV following acute RE, it is noteworthy that average cfPWV did significantly differ from baseline at 30 min post‐RE. This observation aligns with findings by Augustine and colleagues in 2018, who investigated cfPWV responses at rest and following acute RE in 18 females across different phases of their menstrual cycle (i.e., early follicular and luteal phase). While their study did not specifically aim to evaluate test‐retest reliability, investigators similarly reported no significant main effect of visit and no significant visit × time interaction following acute RE. Consistent with the present results, they also observed a significant time effect, with cfPWV significantly changing for 30 min following two acute RE bouts [28]. Together, these findings suggest that both the magnitude and temporal patterns of cfPWV responses are preserved across different menstrual phases and repeated exposure to acute RE. However, differences in exercise protocols across studies limit direct comparisons of directionality of cfPWV responses following RE.

While this study was not designed to assess directional changes in cfPWV following RE, we did observe a significant decrease in arterial stiffness at 30 min following both RE bouts. While this decrease was statistically significant, it was not clinically meaningful (i.e., change of at least 1.0 m/s) [44]. Our findings may be due to methodological factors, such as the incorporation of an aerobic warmup and cool down. The ACSM recommends bookending RE sessions with a brief aerobic warmup and cool down [34]. Acute moderate intensity aerobic exercise alone is demonstrated to decrease arterial stiffness [12], and when paired with RE, it may counteract adverse vascular responses [45]. To our knowledge, we are the only investigation to incorporate aerobic exercise at the start and end of the RE bout. However, the extent to which these aerobic components influenced cfPWV responses post‐exercise is only speculative due to the lack of a control condition within the current investigation.

A potential limitation of the study is the lack of control for the menstrual phases. However, research suggests that the menstrual cycle has minimal effect on cfPWV at rest in premenopausal females [46], and at baseline and up to 30 min post‐RE in healthy females [28]. Furthermore, it has been argued that not controlling for the menstrual cycle in studies investigating vascular control may enhance the ecological validity of the findings [47]. In line with our investigation, while we did not control for menstrual cycle phase, cfPWV demonstrated moderate to good reliability at rest and post‐RE in young health females. An additional limitation of the current investigation is the use of a wide range in exercise intensity (i.e., 60%–80%). While this exercise prescription aligns with current guidelines for RE in healthy adults, previous studies have shown that post‐RE vascular responses may vary depending on the exercise intensity [48, 49, 50]. Future investigations should employ a standardized intensity to better understand the effects of specific RE loads on vascular responses. Moreover, this study was not designed to assess the mechanisms or clinical implications of directional changes in cfPWV. Future work should investigate whether transient changes in cfPWV reflect acute vascular adaptations or measurement variability.

## 5 Conclusion

In summary, our study establishes that cfPWV post‐RE demonstrate consistent responses across acute RE bouts. Moreover, cfPWV demonstrates moderate to good reliability pre‐ and post‐acute RE for up to an hour. We illustrate that post‐RE measurements of cfPWV exhibit a similar level of reliability as measures taken in resting conditions. These findings endorse the utilization of cfPWV measurements in healthy college‐aged females at rest and post‐RE. Further research is needed to ascertain the directional response of cfPWV following acute RE.

## Author Contributions

K.S. and K.C. designed the study. W.B., K.S., and K.C. performed experiments. W.B., and K.S. analyzed the data. W.B. and K.C. interpreted the data. W.B., K.C. drafted the article. All authors read, edited, and approved the final version of the manuscript.

## Conflicts of Interest

The authors declare no conflicts of interest.

## Transparency Statement

The corresponding author, Katharine Dianne Currie, affirms that this manuscript is an honest, accurate, and transparent account of the study being reported; that no important aspects of the study have been omitted; and that any discrepancies from the study as planned (and, if relevant, registered) have been explained.

## Data Availability

The data used in this article will be shared on reasonable request to the corresponding author.
